# Advances in Fruit Aroma Volatile Research

**DOI:** 10.3390/molecules18078200

**Published:** 2013-07-11

**Authors:** Muna Ahmed Mohamed El Hadi, Feng-Jie Zhang, Fei-Fei Wu, Chun-Hua Zhou, Jun Tao

**Affiliations:** College of Horticulture and Plant Protection, Yangzhou University, Jiangsu Key Laboratory of Crop Genetics and Physiology, Yangzhou 225009, China; E-Mails: munaelhadi@hotmail.com (M.A.M.E.); lolita318@163.com (F.Z.); parent0350@163.com (F.W.); taojun@eyzu.edu.cn (J.T.)

**Keywords:** fruit, aroma volatiles, pre & postharvest, biosynthetic pathways

## Abstract

Fruits produce a range of volatile compounds that make up their characteristic aromas and contribute to their flavor. Fruit volatile compounds are mainly comprised of esters, alcohols, aldehydes, ketones, lactones, terpenoids and apocarotenoids. Many factors affect volatile composition, including the genetic makeup, degree of maturity, environmental conditions, postharvest handling and storage. There are several pathways involved in volatile biosynthesis starting from lipids, amino acids, terpenoids and carotenoids. Once the basic skeletons are produced via these pathways, the diversity of volatiles is achieved via additional modification reactions such as acylation, methylation, oxidation/reduction and cyclic ring closure. In this paper, we review the composition of fruit aroma, the characteristic aroma compounds of several representative fruits, the factors affecting aroma volatile, and the biosynthetic pathways of volatile aroma compounds. We anticipate that this review would provide some critical information for profound research on fruit aroma components and their manipulation during development and storage.

## 1. Introduction

Fruit quality includes both its preharvest development, such as changes in colour, flavor, and texture as fruits develop, grow, and ripen, as well as its maintenance following harvest as the perishable tissues senesce [1]. Flavor consists both of the perception in mouth (sweetness, acidity or bitterness) and on the odor, produced by several volatile compounds. All plants are able to emit volatile organic compounds (VOCs) and the content and composition of these molecules show both genotypic variation and phenotypic plasticity [2]. As aroma is one of the most appreciated fruit characteristics, volatile flavor compounds are likely to play a key role in determining the perception and acceptability of products by consumers. Identification of key volatile flavor metabolites that carry the unique character of the natural fruit is essential, as it provides the principal sensory identity and characteristic flavor of the fruit [3].

Aroma is a complex mixture of a large number of volatile compounds, whose composition is specific to species and often to the variety of fruit [4,5]. Although different fruits often share many aromatic characteristics, each fruit has a distinctive aroma that depends upon the combination of volatiles, the concentration and the perception threshold of individual volatile compounds [6]. The most important aroma compounds include amino acid-derived compounds, lipid-derived compounds, phenolic derivatives, and mono- and sesquiterpenes [5]. Although fruit aroma is generally a complex mixture of a wide range of compounds, volatile esters often represent the major contribution in apple (*Malaus domestica* Borkh.) and peach (*Prunus persica* L.) [7,8].

As an important trait of fruit quality, aroma has gained increasing attention in recent years. With the fast development of science and technology especially the application of the GC-MS and other analytical apparatus, progress in aroma research has been made in several fields [9]. In this review, we summarized the composition of fruit aroma and the characteristic aroma compounds of several representative fruits at first, then the factors affecting aroma volatile were discussed, and finally the biosynthetic pathways of volatile aroma compounds were summarized.

## 2. Aroma Volatile Composition and Their Biological Characteristic of Major Fruits

Most fruits produce significant numbers of volatile compounds as indicators of fruit ripening. Many of these volatile compounds are produced in trace amounts, which are below the thresholds of most analytical instruments, but can be detected by human olfaction [10]. Volatiles can be classified as primary or secondary compounds, indicating whether they were present in intact fruit tissue or produced as a result of tissue disruption [11]. It should be pointed out that analysis of volatiles from either intact or disrupted fruit tissues will influence the aroma profiles and final aroma interpretation. The volatile profiles of fruit are complex and vary depending on the cultivar, ripeness, pre-and post-harvest environmental conditions, fruit sample (either intact fruit, slices, or homogenized samples), and analytical methods utilized [12,13]. Aroma compounds are often only released upon cell disruption when previously compartmentalized enzymes and substrates interact [14]. Some aroma compounds are bound to sugars as glycosides or glucosinolates. Glycosides of aroma compounds in fruit are mainly *O*-*β*-D-glucosides and *O*-diglycosides, but triglycosides have also been identified [15]. The proportion of glycosidically bound volatiles is usually greater than that of free volatiles, making them an important potential source of flavor compounds. The odorous aglycones may be released from the sugar moiety during maturation, processing and storage, or by the action of enzymes, acids or heat [16].

### 2.1. Classification of Volatile Compounds in Fruit Flavor

Various types of fresh fruits produce distinct volatile profiles. Flavor volatiles are derived from an array of compounds including phytonutrients such as fatty acids, amino acids, carotenoids, phenols and terpenoids [10]. Fruit volatile compounds are mainly comprised of diverse classes of chemicals, including esters, alcohols, aldehydes, ketones, lactones, and terpenoids. However, some sulfur compounds, such as *S-*methyl thiobutanoate, 3-(methylthio) propanal, 2-(methylthio) ethyl acetate, 3-(methylthio) ethyl propanoate, and 3-(methylthio) propyl acetate, also contribute to the flavor of fruit such as melons (*Cucumis melo* L.) [9]. Bound volatiles are recognized as a potential source of aroma compounds in fruits such as kiwifruit (*Actinidia* *deliciosa*) [17]. Although an overwhelming number of chemical compounds have been detected as volatile compounds in fresh fruit, only a fraction of these compounds have been identified as impact components of fruit flavor based on their quantitative abundance and olfactory thresholds [18]. Many C_10_ monoterpenes and C_15_ sesquiterpenes compose the most abundant group of compounds present in the aroma profile. In some cases, these are also the key compounds determining the characteristic aroma. For example, the terpenoids *S*-linalool, limonene, valencene and *β*-pinene are key aroma compounds of strawberry* (Fragaria x ananassa*), koubo (*Cereus peruvianus* L.) and citrus (*Citrus* sp.) [19,20,21]. Volatile terpenoid compounds, potentially derived from carotenoids, are important components of flavor and aroma in many fruits. Of particular interest are a group of terpenoid flavor volatile compounds generally present at relatively low levels but possessing strong effects on the overall human appreciation. Among these are *β*-ionone, geranylacetone (6,10-dimethyl-5,9-undecadien-2-one), pseudoionone (6,10-dimethyl-3,5,9-undecatrien-2-one), *β*-cyclocitral, geranial, theaspirone, *α*-damascenone and *β*-damascenone. Their structures reveal an isoprenoid-based origin, and they were long assumed to be the products of the oxidative cleavage of carotenoids [22].

### 2.2. Volatile Compounds and Their Biological Characteristic of Major Fruits

More than 300 volatile molecules have been reported in fresh apples [23]. The total number, identity and concentration of volatile compounds emitted by ripening apple fruit are cultivar specific [24]. The contribution of each compound to the specific aroma profile of each cultivar depends on the activity and substrate specificity of the relevant enzymes in the biosynthetic pathway, the substrate availability, the odour threshold above which the compound can be detected by smell, and the presence of other compounds [25]. Esters are the most abundant volatile compounds emitted by apple and, together with *α*-farnesene, have been proposed for cultivar classification [26]. Ethyl 2-methyl butanoate, 2-methyl butyl acetate, and hexyl acetate contribute mostly to the characteristic aroma of “Fuji” apples, while ethyl butanoate and ethyl 2-methyl butanoate are the active odor compounds in “Elstar” apples, and ethyl butanoate, acetaldehyde, 2-methyl butanol, and ethyl methyl propanoate in “Cox Orange” [27]. For the “Pink Lady” cultivar, hexyl acetate, hexyl 2-methyl butanoate, hexyl hexanoate, hexyl butanoate, 2-methylbutyl acetate and butyl acetate were prominent within the blend of volatiles produced by fruit throughout maturation [28]. Among fruit tissues, it has been shown that epidermal tissue produces a greater amount of volatiles than internal tissues [29]. This higher capacity for aroma production by the peel has been attributed to either the abundance of fatty acid substrates or the higher metaboltic activity [30,31].

More than 300 volatile compounds have been identified in pear fruit (*Pyrus pyrifolia* Nak.) [32], methyl and hexyl esters of decadienoate are the character-imparting compounds of the European pear [32,33]. Other volatile esters, for example, hexyl acetate, 2-methylpropyl acetate, butyl acetate, butyl butanoate, pentyl acetate, and ethyl hexanoate possess strong pear-like aromas [32]. Ethyl octanoate and ethyl-(*E*)-2-octenoate contribute to sweet or fruity odors in pears, while a high concentration of 2,4-decadienoates in fruit flesh is accepted by consumers [34]. In addition, hexanal, 2-methylpropyl acetate, ethyl acetate, hexyl acetate, 3-methylbutyl-2-methyl butanoate, ethyl butanoate, and butanol are identified as impact volatiles in “Conference” pears [35].

Although the melon aroma has been extensively investigated and more than 240 volatile compounds have been identified in different varieties [36], the literature is lacking in quantitative data. Numerous compounds of different degrees of volatility, especially those containing a C_9_ straight chain, are the major determinants of melon fruit quality perceived by consumers. These compounds are strongly dependent on the variety and physiological behavior of the fruit; in fact, fresh climacteric melons such as cantaloupe have greater aroma intensity and a shorter shelf life than less climacteric melons such as honeydew [37]. In climacteric aromatic melon varieties, volatile esters are prominent, together with sulphur-containing aroma compounds, sesquiterpenes, norisoprenes, short-chain alcohols, and aldehydes [38]. Volatile esters, mainly acetate derivatives such as ethyl 2-methyl propyl acetate and 2-methyl butyl acetate are dominant with 37% of the total volatile profile [39]. In addition, lower amounts of lactones, sulfur compounds [such as (methylthio) acetate, 2-(methylthio) ethyl acetate and 3-(methylthio) propyl acetate], short-chain alcohols and aldehydes compose the complex mixture of volatile compounds [40,41,42]. Non-aromatic varieties often have much lower levels of total volatiles, and lack the volatile esters [43]. Volatiles derived from amino acids are major contributors to melon aroma [44,45]. Both aromatic and non-aromatic varieties possess amino acid derived volatiles. In the aromatic varieties these volatiles are mostly esterified and their levels are usually higher than in the non-aromatic varieties. In the non-aromatic varieties they occur as aldehydes and alcohols.

Strawberry has one of the most complex fruit aromas, made up of approximately 350 volatile compounds [5,46]. The furanones 2,5-dimethyl-4-hydroxy-3(2*H*)-furanone (furaneol) and its methyl derivative 2,5-dimethyl-4-methoxy-3(2*H*)-furanone (mesifurane) are considered the dominating aroma compounds [47]. They contribute to the typical caramel-like, sweet, floral and fruity aroma. Esters, which are the most important group of strawberry aroma compounds, cover 90% of the total number of volatiles in ripe strawberry fruit [47]. Among the major esters are methyl and ethyl butanoate, butyl acetate, methyl and ethyl hexanoate. Other key aroma compounds are linalool, *γ*-decalactone and 2,3-butanedione though some of these key compounds tend to be cultivar-specific [47,48]. Finally, aldehydes and alcohols such as hexanal, *trans-*2-hexenal and *cis*-3-hexen-1-ol are important for the green, unripe notes in strawberry aroma. Their concentrations are also cultivar and ripeness dependent [47].

Although more than 250 volatile components have been identified in banana [49], the banana fruity top notes are from volatile esters, such as isoamyl acetate and isobutyl acetate [50]. The concentrations of acetates and butanoates increased during ripening of banana fruit [49]. In addition, isoamyl alcohol, isoamyl acetate, butyl acetate, and elemicine were detected by olfactometric analyses as characteristics of banana odor [51]. The main volatile compounds found in “Cavendish” banana were (*E*)-2-hexenal and acetoin, in “Plantain”: (*E*)-2-hexenal and hexanal, and in “Frayssinette”: 2,3-butanediol and two diastereomeric solerols. The most abundant aglycones were 3-methyl butanol, 3-methyl butanoic acid, solerol (two diastereomers) and acetovanillone. This compound, rarely identified in fruits, was detected for the first time in conjugated volatile compounds of fruits. The abundance of these two diastereomer in the extracts of “Frayssinette” seemed to be characteristic of this variety of banana [52].

Citrus volatiles have been extensively examined over the past several decades. Esters are important as they are responsible for the flavor characteristic [27].While the major esters are ethyl esters of C_3_ to C_4_ organic acids, linalool is by far the most important alcohol. However, ketones, carvone, diacetyl, and acetoin are off-flavors. Volatile compounds in citrus fruits accumulate in oil glands of flavedo and in the oil bodies of the juice sacs. Over 100 volatile compounds have been identified for the first time in the juice of four varieties of citrus (Powell Navel orange (*Citrus sinensis* L.), Clemenules mandarine (*C.* *reticulate* Blanco.), Fortune mandarine (*C.* *reticulate* Blanco.) and Chandler pummelo (*C.* *maxima* Merr.)). The differences in the volatile profile in citrus juice are mainly quantitative, and only a few compounds are variety-specific. In Chandler the most characteristic volatiles were principally aliphatic aldehydes, sesquiterpenes such as nootkatone and monoterpenes such as 2-carene. Powell Navel orange showed the highest levels of esters such as nonyl acetate and of monoterpenes such as 3-carene. Clemenules showed the highest levels of ketones 3-pentanone and *β*-ionone and Fortune showed the highest levels of some acetate esters such as ethyl and propyl acetate, this latter almost Fortune-exclusive [53]. Moreover, a total of 58 volatile components were identified and quantified in Dortyol yerli orange juice, terpenes quantitatively and qualitatively represent the main group of the volatile fraction. D,L-limonene was the major terpene component, followed by valencene. After terpenes, terpenols were clearly the dominant constituents. Linalool, terpinen-4-ol and *α*-terpineol were the most abundant among the terpenol compounds. In terms of aroma contribution to orange juice, 12 compounds were prominent based on the odour activity values (OAVs). The highest OAV values were recorded for ethyl butanoate, nootkatone, linalool and *D,L*-limonene [54]. On the other hand, limonene was the most abundant monoterpene hydrocarbon in both the peel and juice of three major Japanese sour citrus cultivars, Yuzu (*C. junos* Sieb. ex Tanaka), Sudachi (*C* *sudachi* Hort. ex Shirai) and Kabosu (*C.* *sphaerocarpa* Hort. ex Tanaka), followed by *γ*-terpinene and *β*-phellandrene in Yuzu and Sudachi and by myrcene in Kabosu. Mintsulfide was newly identified in juice extracts from Yuzu and Kabosu. Among the oxygenated components, linalool was the most abundant in both the peel and juice of Yuzu and the peel of Sudachi, while both the peel and juice of Kabosu revealed the presence of high quantity of saturated aliphatic aldehydes. Wine lactone and rose oxide were identified in all the extracts, which have not been previously reported to occur in these citrus cultivars. With regard to linalool, the *R*-enantiomer was predominant in Yuzu and the *S*-enantiomer in Sudachi and Kabosu [55]. In other citrus species, such as *C.* *natsudaidai* other terpenoids are less abundant, but they exert a profound effect on aroma. These include *γ*-terpinene, *β*-phellandrene, mycerene, and *α*-pinene [20,56].

Mango (*Mangifera indica* L.) possesses a very attractive flavor characteristic. More than 270 aroma volatile compounds in different mango varieties have been identified in free form [57]. However, application of distillation extraction in combination with active odor value (aroma threshold) technologies exhibits that monoterpenes such as *α-*pinene, myrecene, *α*-phelladrene, *σ*-3-carene, *p*-cymene, limone and terpinolene, esters including ethyl-2-methyl propanaote, ethyl butanoate, as well as (*E,Z*)-2,6-nonadienal, (*E*)-2-nonenal, methyl benzoate, (*E*)-*β*-ionone, decanal, and 2,5-dimethyl-4-methoxy-3(2*H*)-furanone are the most important compounds contributing to mango flavor [58]. Generally, terpene hydrocarbons are the major class of compounds in New World mangoes, with contents ranging from 16–90%. 3-Carene is the major compound in most New World mango cultivars, with limonene, *β-*ocimene, myrcene and *α*-terpinolene having some importance in some cultivars. Sesquiterpene hydrocarbons may also be present in amounts as high as 10% in some cultivars. There is a large variation in the quality and quantity of alcohols, ketones, and esters in mangoes, especially those of the Old World varieties. Those compounds, together with esters, are responsible for much of the characteristic aroma of Old World mangoes [59]. Terpene hydrocarbons were the major volatiles of nine varieties of Colombian mango, the dominant terpenes being δ-3-carene (“Haden”, “Irwin”, “Manila” and “Tommy Atkins”), α-pinene (“Hilacha” and “Vallenato”), α-phellandrene (“Van Dyke”) and terpinolene (“Yulima”), while no dominant terpene was found in the fruit of “Springfield” [60]. Aroma volatile compounds in the mango fruit have also been reported to be present in the glycosidically-bound form [61].

Peach fruit volatiles have been extensively studied, and more than 100 compounds were identified [62,63,64], among them, C_6_ aldehydes and alcohols offer the green-note aroma, while lactones and esters are responsible for fruity aromas. Esters such as hexyl acetate and (*Z*)-3-hexenyl acetate are considered as key odorants influencing the flavor quality of peach fruit [65]. Changes in aroma-related volatiles have been reported during peach fruit development and postharvest ripening [66]. Aldehydes tend to decline, while esters increase in the fruit, and postharvest treatments, such as low temperature and controlled atmosphere, have been used to investigate postharvest changes in peach aroma quality [8,67].

To date, more than 200 different volatile compounds have been described in apricots (*Prunus armeniaca* L.) [68]. Six major volatile compounds were identified (hexanal, (*E*)-2-hexenal, linalool, 1-hexanol, ethyl octanoate, and hexyl acetate) in apricot fruit cv. “Modesto”, all of which were previously reported to be major contributors to apricot aroma [69,70]. The most abundant volatile compounds in terms of concentration were aldehydes, mainly hexanal and (*E*)-2-hexenal, and their concentration decreased during ripening with significant differences. Terpenic compounds (i.e. linalool) and alcohols (i.e., 1-hexanol) were produced at a lower concentration than aldehydes, and decreased during ripening with a similar pattern to that observed for aldehydes [69].

Grape (*Vitis vinifera* L.) volatiles include a great number of compounds, among which monoterpenes, C_13_ norisoprenoids, alcohols, esters and carbonyls are found [71]. Grape may be divided into aromatic and nonaromatic varieties. Free terpenols, for example, linalool and geraniol, have been identified as major aroma compounds in both red and white grapes [72]. Octanoic acid and alcohols, particularly 2-phenylethanol, are recognized after crushing [72]. In addition, esters and aldehydes were also reported in “Aleatico” grapes [73]. The most abundant free compounds detected in muscat grape were linalool, geraniol, citronellol, nerol, 3,7-dimethyl-1,5-octadien-3,7-diol (diendiol I) and 3,7-dimethyl-1,7-octadien-3,6-diol (diendiol II). In the glycosidically-bound fraction the major compounds were geraniol, linalool, citral, nerol, citronellol, α-terpineol, diendiol I, diendiol II, *trans*-furan linalool oxide (linaloloxide I), *cis*-furan linalool oxide (linaloloxide II), benzyl alcohol and 2-phenylethanol. Other monoterpenes potentially contributing to Muscat aroma were rose oxide, citral, geraniol, nerol and citronellol [74]. (*E*)-2-hexenal was the most abundant volatile compound in Riesling and Cabernet Sauvignon grapes, and it showed a significant increase in concentration after veraison. Benzene derivatives discriminated ripe Cabernet Sauvignon grapes, whereas monoterpenes and sesquiterpenes discriminated both cultivars pre-veraison with a broader range of terpenes observed in the Cabernet Sauvignon samples compared with the Riesling samples. At veraison, terpene production in both varieties was low, but Riesling grapes produced some terpenes (geraniol and *α*-muurolene) post-veraison. Generally, esters and aldehydes were the major class of compounds from Riesling grapes, while Cabernet Sauvignon showed a greater tendency to form alcohols [75].

Aroma compounds in raspberry (*Rubus idaeus* x *ursinus*) have been studied extensively. At least 200 volatile compounds have been identified in this fruit. Many compounds including raspberry ketone, *α*-ionone, *β*-ionone, linalool, (*Z*)-3-hexenol, geraniol, nerol, *α*-terpineol, Furaneol, hexanal, *β*-ocimene, 1-octanol, *β*-pinene, *β*-damascenone, ethyl 2-methylpropanoate, (*E*)-2-hexenal, heptanal, and benzaldehyde have been identified to contribute raspberry aroma. Among them, *α*-ionone, *β*-ionone, geraniol, nerol, linalool, and raspberry ketone could be particularly important to red raspberry aroma [76]. Twenty-nine volatile compounds were quantified in “Chilliwack”, “Tulameen”, “Willamette”, “Yellow Meeker”, and “Meeker” raspberries, data showed that volatile concentrations varied for different cultivars. Large variations for *α*-ionone, *β-*ionone, geraniol, linalool, and (*Z*)-3-hexenol were observed in different raspberry cultivars. In addition, the volatile compositions in “Meeker” raspberry grown at different locations also varied [77]. Although blackberry (*Rubus laciniata*) has been widely planted, the study of blackberry flavor is still very limited. The early studies focused on the volatile constituents of blackberry, and very diverse compounds have been identified. 2-Heptanol, *p*-cymen-8-ol, 2-heptanone, 1-hexanol, *α*-terpineol, pulegone, 1-octanol, isoborneol, myrtenol, 4-terpineol, carvone, elemicine, and nonanal have all been identified as the major volatiles [78]. In order to understand the aroma differences, the volatile compositions of “Marion” and “Black Diamond” was analyzed using stir bar sorptive extraction-gas chromatography–mass spectrometry (SBSE-GC–MS) and solid phase extraction (SPE)-microvial insert thermal desorption-GC–MS for two growing seasons. Although seasonal variations were present, the overall volatile profile in “Marion” and “Black Diamond” were very similar, but the concentrations of some aroma compounds varied greatly. Odour-activity value (OAV) indicated that furaneol, linalool, *β*-ionone, and hexanal could be most important in “Marion”, while in “Black Diamond”, the most important compounds were linalool, *β*-ionone, furaneol, and 2-heptanol. The major difference between the cultivars for aroma compounds was that “Marion” had higher OAV of furaneol than “Black Diamond”, while “Black Diamond” had higher OAV of linalool than “Marion”. The chemical analysis results matched with the descriptive sensory evaluation that “Marion” had more berry, fruity, strawberry aroma while “Black Diamond” had more floral aroma [79].

A total of 42 volatiles were identified in four southern highbush blueberry (*Vaccinium sp*.)cultivars (“Primadonna”, “Jewel”, “Snowchaser”, and “FL02-40”) , twelve of these volatiles are reported for the first time in highbush blueberries, with 10 being positively identified: (*Z*)-3-hexenal, (*E*,*E*)-2,4-hexadienal, (*E*,*Z*)-2,6-nonadienal, (*E*,*E*)-2,4-nonedienal, methyl 2-methylbutanoate, butyl acetate, 2-methylbutyl acetate, and geranyl acetate. “Primadonna” was characterized by a large amount of esters and C_6_ aldehydes. In contrast, fewer than four esters were found in “FL02-40” and “Snowchaser”, respectively, but they produced more terpenoids than “Primadonna” and “Jewel” [80].

More than 280 volatile compounds have been found in pineapple fruit (*Ananas comosus* L. Merr.) [81]. Esters and hydrocarbons were the major constituents. Octenoic acid, methyl ester, hexanoic acid, octanoic acid and ethyl ester were the specific aromatic components of pineapple fruits. The relative content of volatiles showed significant variations during the fruit developmental period [82]. A total of 11 and 28 volatile compounds were identified in the Tainong No. 4 and No. 6 pineapples, respectively. According to the OAVs, four compounds were defined as the characteristic aroma compounds for the Tainong No. 4 pineapple, including furaneol, 3-(methylthio) propanoic acid methyl ester, 3-(methylthio) propanoic acid ethyl ester and *δ*-octalactone. The OAVs of five compounds including ethyl-2-methylbutyrate, methyl-2-methylbutyrate, 3-(methylthio) propanoic acid ethyl ester, ethyl hexanoate and decanal were considered to be the characteristic aroma compounds for the Tainong No. 6 pineapple [83]. The characteristic aroma compounds in the plup of cayenne pineapple were ethyl 2-methylbutanoate, ethyl hexanoate, 2,5-dimethyl-4-hydroxy-3(2*H*)-furanone (DMHF), decanal, ethyl 3-(methylthio) propionate, ethyl butanoate, and (*E*)-3-ethyl hexenoate; while in core the main compounds were ethyl-2-methyl butanoate, ethyl hexanoate and DMHF. The highest odor units were found to correspond to ethyl 2-methyl butanoate, followed by ethyl hexanoate and DMHF [84].

More than 80 compounds related to fruit aroma have been identified in kiwifruit, with the major components being methyl and ethyl butanoate, (*Z*)- and (*E*)-2-hexenal, hexanal, (*Z*)- and *(E*)-3-hexenol, and methyl benzoate [17]. The volatile profile of eating-ripe “Hort16A” is dominated by ethyl esters and 3-(methylsulfanyl) ethyl propionate has been suggested to contribute to the tropical aroma of non-stored kiwifruit [85,86]. “Hort16A” has been less extensively studied, but it has been noted that an important difference between the aroma profiles of “Hort16A” and “Hayward” is the presence of diverse sulphur compounds in the former [17].

## 3. Factors Influencing Volatile Composition

Due to the complex nature of the volatile profiles, volatile composition is continuously changing in fresh fruit. Many factors affect volatile composition including the genetic makeup of the fruit, its maturity, environmental conditions during production, postharvest handling, and storage. To date we have a limited understanding of how these factors interact to determine the actual volatile composition and resulting flavor of the fruit.

### 3.1. Genetics

Evaluation of volatiles at the germplasm level is useful for future breeding efforts, aimed at improvement of fruit quality, via effects on fruit aroma. The composition and concentration of grape volatiles largely varied with genetic background. In grape cultivars belonging to seven genotypic groups, C_6_ compounds were the dominant volatiles in *Vitis. amurensis* grape, neutral cultivars *V. vinifera* grapes and hybrid grapes between *V. vinife*ra with *V. thunbergi*i or *V. amurensis*. Alcohols and carbonyls were relatively low in all *Vitis* germplasm studied. Terpenoids were abundant in *V. vinifera* with muscat aroma, while esters were dominant in *V. labrusca* and its hybrids with *V. vinifera* or *V. amurensis* [87]. The main aromatic components in three early apple cultivars (“Zaofengtian”, “Vista Bella” and “Liaofu”) belonging to 12 categories were 1-hexanol, (*E*)-2-hexenal, acetic acid, butyl ester, acetic acid, hexyl ester, *etc.*, representing 93.81% of the total aromatic content. *β*-Damascenone and estragole in “Zaofengtian”, which weren’t detected in “Vista Bella” or “Liaofu”, could be unique components to “Zaofengtian” [88]. In strawberry, differences have been observed between cultivated and wild-type varieties with the monoterpene-linalool and the sesquiterpene nerolidol being the most abundant in cultivated varieties, while oleafinic monoterpenes and myrenyl acetate are more important in the wild-type varieties [89,90].

Insertion of the *rin* gene to reduce ethylene production and slow tomato fruit softening, resulted in some deterioration in flavor quality and reduction in flavor volatiles [91]. Transgenic fruit with antisense amino cyclopropanecarboxylic acid (ACC) synthase had lowered levels of many important flavor volatiles [91]. Fruit with antisense pectinmethylesterase had lowered levels of methanol, while those with downregulated phytoene synthase had lowered levels of carotenoid-derived volatiles [91]. The transformed melon plants displayed a range of decreased alcohol acyltransferase (AAT) activities in the ripening fruit. Therefore, the relative content of volatile esters was reduced, and the average relative content in the transgenic fruit was 78% of the wild-type fruit. However, the relative contents of aldehydes and alcohols increased, the average relative contents were 3.2 times and 2.4 times of those in the wild-type fruit, respectively [92].

### 3.2. Maturity

Many factors including cultivar, cultural practices, ripeness, harvest maturity and postharvest handling can influence the abundance of volatile compounds in fruit. Of these factors, maturity is one of the critical factors to influence the abundance of volatile compounds in fruit [93]. Ideally, fruit should be harvested at optimal eating quality to optimize volatile content for flavor. However, immature fruit are often harvested in order to increase storage and market life and minimize physical damage and disorder expression. Although immature fruit are more successfully stored and transported, flavor is often lacking due to the close relationship between maturity and volatile biosynthesis [94].

In apples, immature fruits produce low quantities of volatiles at harvest, and lose the capability of volatile production during storage more readily than mature fruit [95]. In “Cigaline” and “Chandler” varieties, C_6_ aldehydes and alcohols products of the enzymatic breakdown of unsaturated fatty acids, are major contributors to the flavor of immature fruits in the absence of furanones and esters. During fruit ripening, levels of C_6_ compounds decrease drastically with increasd furanone acid lactone and ester production [96]. The enhancement of volatile ester production by “Golden Reinders” apples at late maturity stages may have arisen mainly from greater availability of substrates, thus pointing out the relevance of regulating points located upstream of AAT in the pathway [7]. Similar to apples, harvest maturity plays a pivotal role in volatile development of strawberry fruit. C_6_ aldehydes were identified as the major compounds in immature white fruit, while furanone and esters are present in three quarters or fully red fruit [96]. Flavor volatile development in melons is also closely linked to fruit maturity with the concentration of total volatile compounds increasing linearly with increasing maturity in cantaloupe melons [97]. El-Mageed noted that ethanol, (*Z*)-3-hexanol and (*E*)-2-hexenal, the most abundant volatiles in whole green and ripe “Fuerte” avocados (*Persea Americana* Mill), declined with ripening, while overripe fruit had the highest ester concentration [98]. Pereira reported that the most abundant volatiles present in the headspace of unripe, diced “Simmonds” avocado were sesquiterpenes and hexanal, whereas the amounts of these compounds was greatly reduced by ripening [99]. During maturation time of avocados cv. “Hass” the concentration of hexanal, (*E*)-2-hexenal and 2,4-hexadienal greatly declined in amount, while acetaldehyde, methyl acetate, pentanal, and *β*-myrcene were at higher concentrations in mature fruit and may also have contributed to the overall flavor [100]. In detached “Pluk Mai Lie” papaya fruit, 2-ethyl-1-hexanol was found specifically in green fruit, ethyl octanoate emerged only in fully-ripe fruit. Furthermore, benzyl isothiocyanate was the most abundant volatile present in fruit at every stage except full ripening. The levels of methanol and ethanol sources in fruit increased steadily throughout ripening, with esters formed from ethyl alcohol predominating from the half-ripe through the senescence phases. The alcohol dehydrogenase (ADH) activity in the mesocarp increased dramatically during the early ripening stages, whereas ATT was active throughout ripening [101]. Yang *et al.* [102] reported that all the alcohols and carbonyls, along with most of the C_6_ compounds and terpenoids, were evident before veraison in three different flavour table-grapes, “Jingxiu”, “Bimeijia” and “Jingya”, while most of the esters were detected at or after veraison. C_6_ compounds increased in the early period of maturation, and then decreased. Most alcohols and carbonyls tended to continuously decrease during ripening. Except for geraniol, terpenoids increased until maturation, then decreased. Some esters continued to increase after maturation. Principal component analysis showed that terpenoids and esters were the characteristic volatiles of ripe “Bimeijia” and “Jingya” grapes, respectively. “Bimeijia” had the highest terpenoid content at maturity, while “Jingya” continued to accumulate some esters after maturation.

### 3.3. Pre-Harvest Factors

Pre-harvest factors such as sunlight, water availability, fertilization, and chemical applications affect crop growth, and can affect internal quality characteristics of the harvested product, including flavor. Heavy rains prior to harvest dilute flavor compounds in tomatoes (*Solanum lycopersicum* L.). Grape aroma potential was highest in vines under mild water deficit and moderate nitrogen supply. Severe water deficit stress seemed to limit aroma potential, as did nitrogen deficiency [103]. Volatile production of “Golden Delicious” apples was affected by aminoethoxyvinylglycine (AVG) application, especially in the case of esters and alcohols, which presented 59% and 33% lower values in AVG-treated apples at the end of storage. A higher concentration of aldehydes was observed at harvest time in AVG-treated apples. Higher correlations between ethylene production and aldehydes and alcohols were found in control apples than in AVG-treated apples. However, AVG treatment negatively affects the production of some volatile compounds [104].

### 3.4. Postharvest Handling

Various techniques are used to extend the shelf-life of fruits after harvest. These storage techniques and treatments involve cold, heat, irradiation, different storage atmospheres, and chemical applications. These postharvest handlings can also affect the aroma components and concentrations.

#### 3.4.1. Temperature

Storage temperature is a fundamental factor affecting the flavor of fruits. Changes in storage temperatures had no major effects on aroma volatile contents in chilling-tolerant “*Or*” mandarins; however, in chilling-sensitive “Odem” mandarins, storage at 2 °C caused accumulation of 13 volatiles, mainly terpenes and their derivates, whereas storage at 8 °C resulted in decreases of six volatiles, comprising five terpenes and one terpene derivative [105]. Production of volatiles was markedly influenced by storage temperature and time in melting flesh peach “Hujingmilu” fruit. In general, fruit at 5 °C were sensitive to chilling injury and had the lowest levels of volatile compounds, especially fruity note volatiles such as esters and lactones [67]. Refrigeration of tomato induced changes in levels of 3-methylbutanal, linalool, guiacol, hexanol, *trans*-2-hexenal and *trans*-3-hexenol, and some of these alterations may be explained by a decrease in ADH enzyme activity [106]. During cold storage at 4 °C, acetate esters declined and non-acetate esters increased in fresh-cut cantaloupe and honeydew melons. A significant shift in the ratio between acetate and non-acetate esters can be seen even after 2 days in storage. The recycling of esters during storage of fresh-cut melons may lead to an imbalance of characteristic volatiles [107]. Both chilling and heating of tomatoes reduced C_6_ aldehyde and alcohol aroma volatiles immediately after treatment, and the levels of aldehydes did not fully recover after 4 days at 20 °C. Chilling-induced inhibition of C_6_ volatile production may be due to down-regulation of gene expression, and subsequent reduction of hydroperoxide (HPL) and ADH enzyme activities in the oxylipin pathway. Heating-inhibition of C_6_ volatile production, however, does not appear to be due to down-regulation of gene expression, but HPL and ADH activities were briefly suppressed [108].

#### 3.4.2. Storage Atmosphere

Lowering O_2_ and raising CO_2_ can maintain the quality of many fresh fruits for extended periods. However, exposure of fresh product to O_2_ levels below their tolerance level can increase anaerobic respiration and lead to the development of off-flavor. Storage of fruit under controlled atmosphere (CA) conditions can reduce the capacity of several fruit to produce ethylene and alter production of aroma volatiles [109]. Storage of peach fruit cv “Rich Lady” for 15 days, under 3% O_2_ + 10% CO_2_ at 2 °C, improved juiciness, sweetness, perception of flavor, emission of aroma volatile compounds and sensory acceptance in comparison with fruit stored in cold air [110]. Decreasing O_2_ levels and increasing CO_2_ levels of “Titania” blackcurrants retarded the capacity of 3-week stored fruit to synthesize terpenes. Differential changes among the various groups of terpenes were more important, where terpene alcohols reached a peak in 6-week air-stored fruit, and storing berries under a high CO_2_ level (18 kPa) and/or decreasing O_2_ level (2 kPa) resulted in lower biosynthesis of these alcohols compared to control fruit. CA storage conditions led to a transitory reduction in the emission of alcohols but a recovery was recorded with prolonged storage. Non-terpene esters differed greatly in storage, in particular the ester ethyl butanoate. Air-stored fruit at both sampling dates synthesized significantly higher amounts of esters than freshly harvested fruit but a significant decline was observed for branched butyl substances (2-methyl butyl butanoate) after 6 weeks storage [111].

Use of packaging and edible coatings can create a modified atmosphere (MA) with reduced O_2_ and elevated CO_2_ levels, similar to that of CA. Use of edible coatings affects flavor and the level of volatile flavor compounds in citrus, apple and mango fruit [112,113,114]. The coating barrier probably induced anaerobic respiration and the synthesis of ethanol and acetaldehyde, and entrapped volatiles, including ethanol and acetaldehyde [115]. The effects of different edible coatings on mango fruit showed that mango “carnauba” was effective in retarding fruit ripening, retaining fruit firmness, and improving fruit quality attributes including levels of fatty acids and aroma volatiles. Semperfresh and *A. vera* gel (1:1 or 100%) slightly delayed fruit ripening but reduced fruit aroma volatile development. *A. vera* gel coating did not exceed the commercial mango “carnauba” and Semperfresh in retarding fruit ripening and improving aroma volatile biosynthesis [113].

#### 3.4.3. Chemical Application

In addition to CA, other gaseous treatments of fruits and vegetable have been reported. Use of ethylene to synchronize ripening has been practiced for years on banana and tomato, and for degreening of citrus. Ethylene treatment of tomato fruit alters volatile levels [116]. 1-methylcyclopropene (1-MCP) treatment of peach fruit “Tardibelle” altered the supply of alcohol and acyl-CoA precursors, leading to significant changes in the emission of some volatile esters, particularly of the straight-chain type [8].

Other chemical treatments of fresh product may also affect flavor. Calcium treatment of fruit is a widely used practice aimed mainly at avoiding the development of bitter pit. Calcium treatment of commercially mature “Golden Reinders” apples notably enhanced the production of aroma volatile compounds after mid-term storage under air and, to a lesser extent, under standard CA. Aroma volatile production was severely depleted in ultra-low oxygen atmosphere stored samples, and calcium treatment could not overcome this inhibition [117]. Methyl jasmonate (MJ) alone and in conjunction with ethanol, is able to modify the biochemical pathways of volatile compounds [118]. Reports in the literature have described the impact of MJ on biosynthesis of volatile compounds in both climacteric fruit, such as apples and non-climateric fruit, such as strawberries [119,120,121,122]. The application of MJ may avoid undesirable alterations in the volatile fraction occurring postharvest and during storage, as well as minimizing aroma losses. Postharvest MJ treatments in combination with ethanol on the formation of aroma constituents in berryfruit (raspberries, strawberries and blackberries) showed different effects according to berry species. In contrast to raspberries, which exhibited a significant decline in the total amount of volatiles after treatment, a significant enhancement of total volatile compounds was observed in strawberries, while no significant effect was found in blackberries. Esters and terpene compounds responded similarly in strawberries and blackberries suggesting similarity in the biochemistry of their aroma synthesis. In contrast, raspberry volatile compounds showed a different pattern, reflecting different biosynthetic pathways for aroma formation in raspberry. The natural volatile compounds, MJ and ethanol, seemed to have either promoting effects on the formation of the (−)-enantiomers of chiral terpenes and ionones or inhibitory effects on the synthesis of the corresponding (+)-enantiomers [123].

## 4. Volatile Aroma Compounds Biosynthestic Pathways and Related Enzymes

As volatiles are comprised of at least five chemical classes, there are several pathways involved in volatile biosynthesis. These have not been fully described but appear to be common for different fruits. Volatiles important for aroma and flavor are biosynthesized from amino acids, membrane lipids and carbohydrates [4]. Although volatile compounds are synthesized via a few major biochemical pathways, various forms of enzymatic modifications such as hydroxylations, acetylations, and methylations, add to the diversity of emitted volatiles by increasing their volatility at the final step of their formation [124,125]. An important step in the biosynthetic pathway of aroma compounds is the availability of primary precursor substrates, including fatty acids and amino acids, which are highly regulated during fruit development in terms of amount and composition [126].

### 4.1. Fatty Acids Pathway

Fatty acids are major precursors of aroma volatiles in most fruit [4]. Fatty acid-derived straight-chain alcohols, aldehydes, ketones, acids, esters and lactones ranging from C_1_ to C_20_ are important character-impact aroma compounds that are responsible for fresh fruit flavors with high concentrations, and are basically formed by three processes: *α*-oxidation, *β*-oxidation and the lipoxygenase pathway [127]. Aroma volatiles in intact fruit are formed via the *β-*oxidation biosynthetic pathway, whereas when fruit tissue is disrupted, volatiles are formed via the lipoxygenase (LOX) pathway [128]. Nevertheless, some studies suggest that increasing availability of fatty acid, along with higher membrane permeability, during fruit ripening might allow the LOX pathway to become active in intact plant tissue and to function as an alternative to *β*-oxidation [30]. Many of the aliphatic esters, alcohols, acids, and carbonyls found in fruits are derived from the oxidative degradation of linoleic and linolenic acids [16]. In addition, some of the volatile compounds derived from enzyme-catalyzed oxidative breakdown of unsaturated fatty acids may also be produce by autoxidation [129]. Autoxidation of linoleic acid produces 9,13-hydroperoxides, whereas linolenic acid also produces 12,16-hydroperoxides [27]. Hexanal and 2,4-decadienal are the primary oxidation products of linoleic acid, while autoxidation of linolenic acid produces 2,4-heptadienal as the major product. Further autoxidation of these aldehydes leads to the formation of other volatile products [129].

#### 4.1.1. *β*-Oxidation

Although the degradation of straight chain fatty acids by α and *β*-oxidation is a major process for the formation of flavor molecules in all organisms, the specific pathways in plants are not well understood. Baker *et al.* described varied roles for this pathway in relation, not only to fatty acid catabolism but also, to amino acid metabolism and biosynthesis of hormonal compounds [130]. *β*-Oxidation results in successive removal of C_2_ units (acetyl CoA) from the parent fatty acid. The detailed mechanisms of conventional *β*-oxidation are well established [131]. Sanz *et al.* reported that β-oxidation of fatty acids is the primary biosynthetic process providing alcohols and acyl coenzyme A (CoAs) for ester formation [4]. Fatty acid acyl-CoA derivatives are converted to shorter chain acyl CoAs by losing two carbons in every round of the *β*-oxidation cycle, requiring flavinadenine dinucleotide (FAD), nicotinamide adenine dinucleotide (NAD), and free CoA. Acyl CoAs are reduced by acyl CoA reductase to aldehyde that in turn is reduced by ADH to alcohol for use by AAT to produce esters [132]. Pear and apple aromas have been two classical examples of volatile formation through the *β*-oxidation pathway [133].

The biosynthesis of lactones, key aroma components in fruits such as peach and nectarine (γ-decalactone and γ-dodecalactone), pineapple (δ-octalactone), or coconut (*Cocos nucifera* L.) (γ-octalatcone), is also associated with the β-oxidation pathway [134]. In fact, most hypotheses on lactone biosynthesis in fruits put in contact the two major pathways producing aroma compounds from fatty acid, *β*-oxidation, and LOX [4]. Despite the importance of these compounds in fruit aroma, there is a lack of enzymatic studies in fruits, and microorganisms serve as a model for studying lactone biosynthesis [135].

#### 4.1.2. Lipoxygenase (LOX)

The metabolism of polyunsaturated fatty acids, via the first LOX-catalyzed step and the subsequent reactions, is commonly known as the LOX pathway. Saturated and unsaturated volatile C_6_ and C_9_ aldehydes and alcohols are important contributors to the characteristic flavors of fruits, vegetables and green leaves. The short-chain aldehydes and alcohols are produced by plants in response to wounding and play an important role in the plants defense strategies and pest resistance [136,137]. At least four enzymes are involved in the biosynthetic pathway leading to their formation: LOX, HPL, 3(Z), 2(*E*)-enal isomerase and ADH. When fruit are homogenised, linoleic and linolenic acid are oxidised to various C_6_ and C_9_ aldehydes [138]. In intact fruit, enzymes in the LOX pathway and their substrates have different subcellular locations, preventing formation of volatile compounds [4]. During ripening, cell walls and membranes may become more permeable, allowing the LOX pathway to become active without tissue disruption [4]. The LOX biosynthetic pathway has the potential to provide substrates for ester production [139]. If the LOX biosynthetic pathway were active during ripening, it would act as an alternative to β-oxidation of fatty acids.

**Scheme 1:**
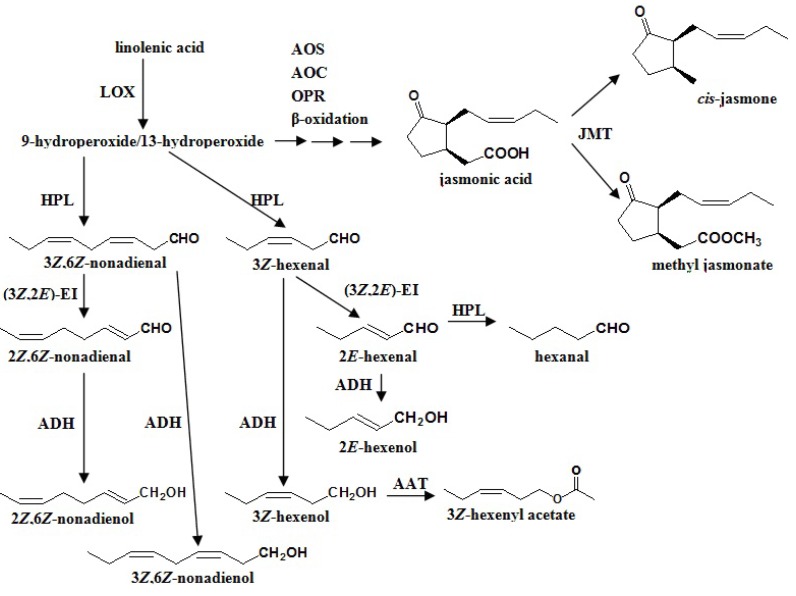
Linolenic acid-derived flavor molecules.AAT, alcohol acyl CoA transferase; ADH, alcohol dehydrogenase; AER, alkenal oxidoreductase; AOC, allene oxide cyclase; AOS, allene oxide synthase; HPL, hydroperoxide lyase; JMT, jasmonate methyltransferase; LOX, lipoxygenase; OPR, 12-oxo-phytodienoic acid reductase; 3*Z*,2*E*-EI, 3*Z*,2*E*-enal isomerase.

As shown in Scheme 1, volatile fatty acid derivatives such as *trans-*2-hexenal, *cis*-3-hexenol and methyl jasmonate are derived from C_18_ unsaturated fatty acids including linoleic acid or linolenic acid, which undergo dioxygenation in a reaction catalyzed by LOX [140]. These enzymes can catalyze the oxygenation of polyenoic fatty acids at C_9_ or C_13_ positions yielding two groups of compounds, the 9-hydroperoxy and the 13-hydroperoxy derivatives of polyenoic fatty acids. These derivatives can be further metabolized by an array of enzymes, including allene oxide synthase (AOS) and HPL, which represent two branches of the lipoxygenase pathway yielding volatile compounds. In the AOS branch of the lipoxygenase pathway, 13-hydroxyperoxy linolenic acid is converted to 12,13-epoxyoctadecatrienoic acid by AOS [140]. A series of subsequent enzymatic reactions leads to the formation of jasmonic acid, which can in turn be converted to the volatile ester, methyl jasmonate, by the enzyme jasmonic acid carboxyl methyltransferase [141]. In the HPL branch of the LOX pathway, the oxidative cleavage of hydroperoxy fatty acids through the action of HPL leads to the formation of short chain C_6_ or C_9_ volatile aldehydes (e.g., 3-hexenal or 3,6-nonadienal) and the corresponding C_12_ or C_9_*ω-*fatty acids (e.g., 12-oxo-dodecenoic acid or 9-oxononanoic acid). HPL C_6_ aldehyde products can be further converted to their isomers by spontaneous rearrangement by alkenal isomerases, or reduced to alcohols by the action of ADH [142].

### 4.2. Amino Acid Pathway

**Scheme 2:**
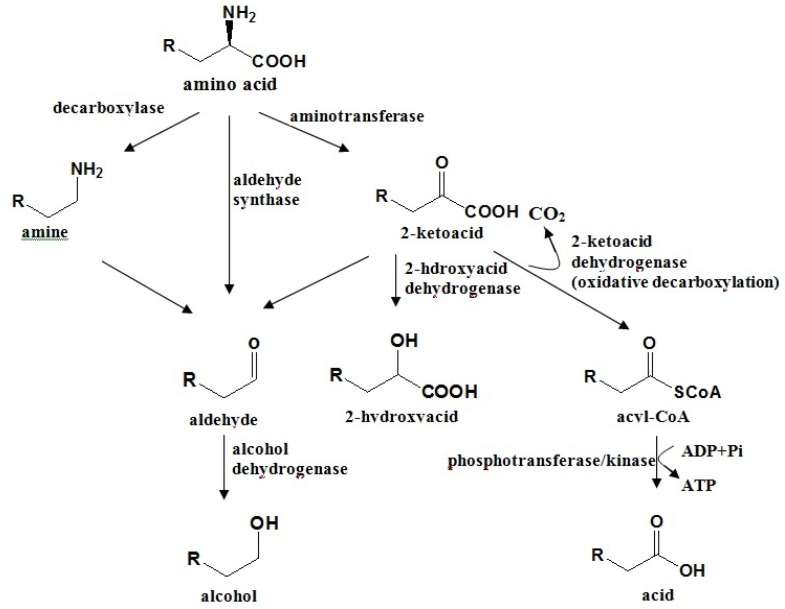
Biosynthetic routes for amino acid degradation to volatiles in plants and microorganisms.

Amino acid, such as alanine, valine, leucine, isoleucine, phenylalanine and aspartic acid, are also involved in aroma biosynthesis in fruit as direct precursors, and their metabolism is responsible for the production of a broad number of compounds, including alcohols, carbonyls, acids and esters [4,143]. Most of the information available to date on the biosynthesis of amino acid-derived volatiles in plants is based on precursor feeding experiments with radio-labeled, stable-isotope-labeled, or unlabeled precursors. The general scheme of biosynthesis is thought to proceed in a similar way as that in bacteria or yeast, where these pathways have been studied more extensively [144,145]. Amino acids can undergo an initial deamination or transamination leading to the formation of the corresponding *α*-keto acid (Scheme 2). Subsequent decarboxylation followed by reductions, oxidations and/or esterifications give rise to aldehydes, acids, alcohols and esters [16]. Branched chain volatile alcohols, aldehydes and esters in fruits such as banana, apple, strawberry and tomato arise from the branched chain amino acids leucine, isoleucine and valine [10,146,147,148]. These amino acids can also be the precursors of acyl-CoAs, which are used in alcohol esterification reactions catalyzed by AATs. Indeed, isoleucine could give rise to 3-methylbutanol and 2-methylbutyryl-CoA, both used in an esterification reaction to yield the ester 3-methylbutyl 2-methylbutanoate in banana [149]. Methionine could be the precursor of sulphur-containing volatiles such as dimethyldisulfide and volatile thioesters [18]. In strawberry, it has been suggested that alanine serves as a precursor for volatile ethyl esters, which can be produced by AAT [149,150].

### 4.3. Terpenoids Pathway

**Scheme 3:**
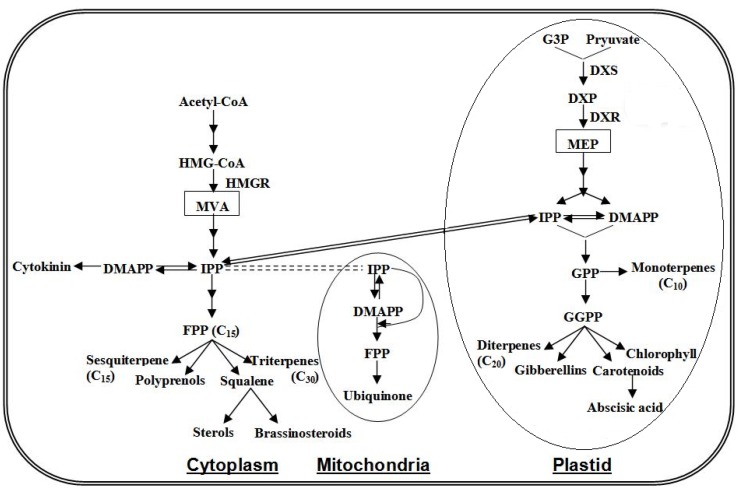
The biosynthesis pathway of isoprenoids in plant cell. DMAPP, dimethylallyl diphosphate; DXP, 1-deoxy-D-xylulose-5-phosphate; DXS, DXP synthase; DXR, DXP reductoisomerase; FPP, farnesyl diphosphate; G3P, glyceraldehyde 3-phosphate; GPP, geranyl diphosphate; GGPP, geranyl geranyl diphosphate; HMGR, HMG-CoA reductase; IPP, isopentenyl diphosphate; MEP, methylerythritol phosphate; MVA, mevalonic acid.

The terpenoids compose the largest class of plant secondary metabolites with many volatile representatives. Hemiterpenes (C_5_), monoterpenes (C_10_), sesquiterpenes (C_15_), homoterpenes (C_11_ and C_16_), and some diterpenes (C_20_) have a high vapor pressure allowing their release into the atmosphere. As shown in Scheme 3, terpenoids are derived from the universal C_5_ precursor isopentenyl diphosphate (IPP) and its allylic isomer dimethylallyl diphosphate (DMAPP), which in higher plants are generated from two independent pathways located in separate intracellular compartments. In cytosol, IPP is derived from the long-known mevalonic acid (MVA) pathway that starts with the condensation of acetyl-CoA [151]. In plastids, IPP is formed from a MVA-independent pathway (or MEP pathway) with pyruvate and glyceraldehydes 3-phosphate as direct precursors and methylerythritol phosphate (MEP) as the key intermediate [152]. Initial research indicated that the cytosolic IPP serves as a precursor of farnesyl diphosphate (FPP) for sesquiterpenes and triterpenes, whereas the plastidial IPP provides the precursors for geranyl diphosphate (GPP) and GGPP for mono-, di-, and tetra-terpenes. However, cross-talk between these two IPP biosynthetic pathways is prevalent, particularly in the direction from plastids to cytosol [153,154].

In plastids, DMAPP generated from the MEP pathway is used by isoprene synthases for isoprene formation [155,156]. In the cytosol and in plastids, IPP and DMAPP are used by prenyltransferases to produce prenyl diphosphates. In cytosol, the condensation of two molecules of IPP and one molecule of DMAPP catalyzed by the enzyme farnesyl pyrophosphate synthase (FPPS) results in the formation of FPP (C_15_), the natural precursor of sesquiterpenes [157]. In plastids, a head-to-tail condensation of one molecule of IPP and one molecule of DMAPP catalyzed by geranyl pyrophosphate synthase (GPPS) forms GPP (C_10_), the universal precursor of all the monoterpenes [158]. The condensation of one molecule of DMAPP with three molecules of IPP by the action of geranylgeranyl pyrophosphate synthase (GGPPS) yields GGPP, the C_20_ diphosphate precursor of diterpenes [158,159]. Following the formation of the acyclic precursors GPP, FPP, and GGPP, a wide range of structurally diverse cyclic and acyclic monoterpenes, sesquiterpenes, and diterpenes is generated through the action of a large family of enzymes known as terpene synthases/cyclases (TPSs) [160,161]. One of the most outstanding properties of these enzymes is their proclivity for making multiple products from a single prenyl diphosphate substrate [162].

Many of the terpene volatiles are direct products of terpene synthases, while others are formed through alterations of the primary terpene skeletons made by TPSs by hydroxylation, dehydrogenation, acylation, and other reaction types [124]. 3-Hydroxylation of limonene by a P450 enzyme yields *trans*-isopiperitenol, a volatile compound found in mint (*Mentha aquatic*) [163], while 6-hydroxylation of limonene by another P450 enzyme results in the formation of *trans*-carveol, which undergoes further oxidation by nonspecific dehydrogenases to form carvone, a major aroma volatile of caraway fruits (*Carum carvi* L.) [164,165]. Another monoterpene alcohol, geraniol, was also found to be converted to the corresponding aldehyde by dehydrogenases in the glands of sweet basil [166]. On the other hand, an acetylation of geraniol by acetyltransferase generates geranyl acetate, a volatile compound found in the scent of many plant species [167,168]. Recently, reduction of geraniol to *S*-citronellol, the precursor of the potent odorant rose oxide, was confirmed enzymatically in grape mesocarp [169]. Modification reactions are also involved in the formation of terpenoids with irregular acyclic C_16_ and C_11_ carbon skeletons, so called homoterpenes, which are mainly emitted from injured tissues. Although the exact biosynthetic routes to homoterpenes, (*E,E*)-TMTT (C_16_) and (*E*)-DMNT (C_11_) still remain unclear, it is believed that they are derived from geranyl-linalool (C_20_) and (3*S*)-(*E*)-nerolidol (C_15_), respectively, by oxidative degradation possibly catalyzed by cytochrome P450 enzymes [170].

### 4.4. Carotenoid Pathway

Carotenoid and their apocarotenoid derivatives are isoprenoid molecules important for the primary and secondary metabolisms of plants and other living organisms [171]. The oxidative cleavage of carotenoids leads to the production of apocarotenoids and is catalyzed by a family of carotenoid cleavage dioxygenases (CCDs) [172]. Apocarotenoid volatiles are synthesized only at the latest stage of ripening, even though the CCD enzymes are present throughout fruit development [173]. CCDs often exhibit substrate promiscuity, which probably contributes to the diversity of apocarotenoids found in nature [172]. CCDs also differ for their subcellular localization: some, like CCD1, are predicted to be cytosolic; others possess transit peptides for plastid or plastoglobule targeting [174]. In general, the biosynthesis of carotenoid-derived volatile compounds occurs via three steps: an initial dioxygenase cleavage yielding apocarotenoids, followed by enzymatic transformations of these apocarotenoids leading to the formation of polar aroma precursors, and finally acid-catalyzed conversions of these precursors to volatile compounds [22]. However, in some cases a volatile product is the result of the initial dioxygenase cleavage step, as was shown for *β*-ionone in Arabidopsis (*Arabidopsis thaliana*) [175], tomato (*Solanum lycopersicum*) and petunia (*Petunia hybridea*) [172,176].

**Scheme 4:**
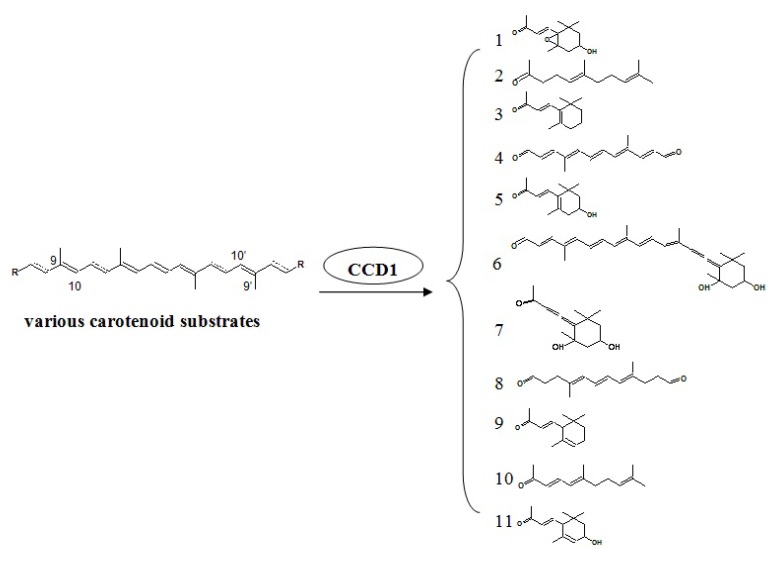
Carotenoids and their degradation products. 1, 5,6-epoxy-3hydroxy-*β*-ionone; 2, geranayl acetone (6,10-dimethyl-5,9-undecadien-2-one); 3, *β*-ionone; 4, C_14_ dialdehyde (4,9-dimethyldodeca-2,4,6,8,10-pentaene-1,12-dial); 5, 3-hydroxy-*β*-ionone; 6, the cleavage product of 9-*cis*-neoxanthin on the 9,10 double bond; 7, the cleavage product of 9-*cis*-neoxanthin on the 9′,10′ double bond; 8, 4,9-dimethyldodeca-4,6,8-trienedial; 9, *α*-ionone; 10, pseudoionone (6,10-dimethyl-3,5,9-undecatrien-2-one); 11, 3-hydroxy-α-ionone.

CCDs exhibit specificity for the double bond that they cleave but many are promiscuous in their substrate choice. In plants, CCD1 and CCD7 cleave the 9,10 double bonds of their respective carotenoid substrates, *At*CCD1 cleaves linear and cyclic carotenoids at the 9,10 and 9′,10′ positions. For example, when β-carotene serves as the substrate, *At*CCD1 produces two C_13_ products (both β-ionone) and a central C_14_ dialdehyde (Scheme 4). CCD7 can cleave both linear and cyclic carotenoid substrates at the 9,10 double bond [177]. Schwartz *et al.* determined that CCD7 cleaves β-carotene asymmetrically, generating a C_13_ ketone (*β*-ionone) and a C_27_ aldehyde (10`-apo-β-carotenal). CCD8 can then cleave the C_27_ aldehyde at its 13,14 double bond [178]. Subsequent data demonstrate, however, that CCD8 can act directly upon carotenoid substrates [179]. In addition, *Bo*LCD from *Bixa orellana* was reported to cleave at the lycopene 5,6 double bond [179], and *Cs*ZCD from *Crocus sativus* was found to cleave at the 7,8 double bond of zeaxanthin [180].

## 5. Conclusions

Fruit aroma is an important indicator to reflects the quality of fruit flavor. Fruits synthesize and emit a large variety of aroma volatile compounds with terpenoids and fatty-acid derivatives the dominant classes. Whereas some volatiles are probably common to almost all fruits, others are specific to only one or a few related fruits. Production of volatiles was markedly influenced by many factors, to date we have a limited understanding of how these factors interact to determine the actual volatile composition and resulting flavor of the fruit. In general, more than one biochemical pathway is responsible for a blend of volatile compounds released from different fruits. Within this final issue, further information about the genes that are involved in the synthesis of aroma volatile should be reviewed.
